# Role of KIT-Positive Interstitial Cells of Cajal in the Urinary Bladder and Possible Therapeutic Target for Overactive Bladder

**DOI:** 10.1155/2011/816342

**Published:** 2011-07-17

**Authors:** Yasue Kubota, Yoshiyuki Kojima, Yasuhiro Shibata, Makoto Imura, Shoichi Sasaki, Kenjiro Kohri

**Affiliations:** Department of Nephro-Urology, Nagoya City University Graduate School of Medical Sciences, Nagoya 467-8601, Japan

## Abstract

In the gastrointestinal tract, interstitial cells of Cajal (ICCs) act as pacemaker cells to generate slow wave activity. Interstitial cells that resemble ICCs in the gastrointestinal tract have been identified by their morphological characteristics in the bladder. KIT is used as an identification marker of ICCs. ICCs in the bladder may be involved in signal transmission between smooth muscle bundles, from efferent nerves to smooth muscles, and from the urothelium to afferent nerves. Recent research has suggested that not only the disturbance of spontaneous contractility caused by altered detrusor ICC signal transduction between nerves and smooth muscle cells but also the disturbance of signal transduction between urothelial cells and sensory nerves via suburothelial ICC may induce overactive bladder (OAB). Recent reports have suggested that KIT is not only a detection marker of these cells, but also may play a crucial role in the control of bladder function. Research into the effect of a c-kit receptor inhibitor, imatinib mesylate, on bladder function implies that KIT-positive ICCs may be therapeutic target cells to reduce bladder overactivity and that the blockage of c-kit receptor may offer a new therapeutic strategy for OAB treatment, although further study will be needed.

## 1. Introduction

Overactive bladder (OAB) syndrome is characterized by urinary frequency and urgency with or without urge incontinence, and is often accompanied by nocturia. In the USA population, 16.5% (16% of men and 16.9% of women) over 18 years of age had symptoms consistent with OAB [1]. The prevalence of this condition increases with age, and OAB significantly impacts health-related quality of life [1–3].

Urgency is the core symptom of the OAB symptom complex, but the underlying mechanisms are not fully understood [4]. OAB symptoms were traditionally considered to result from overactivity of the bladder detrusor muscle. Drake et al. showed model of peripheral autonomous modules and a myovesical plexus in normal and OAB function, and detrusor overactivity (DO) results from exaggerated symptomatic expression of peripheral autonomous activity, resulting from a shift in the balance of excitation and inhibition in smooth muscle modules [5]. On the other hand, there is increasing evidence showing that the urothelium has specialized sensory and signaling properties, and may mediate urgency [6, 7]. In addition, the role of interstitial cells in mediating urgency and the pathophysiology of OAB has recently attracted considerable attention. 

In the gastrointestinal tract, interstitial cells of Cajal (ICC) act as primary pacemaker cells which inject depolarizing currents into neighboring smooth muscles to initiate spontaneous slow waves and corresponding phasic contractions [8], and play a fundamental role in the transmission of signals from enteric neurons to smooth muscle cells [9]. ICCs express the proto-oncogene c-kit, and signaling via the receptor kinase gene product, KIT [10], which is used as an identification marker of ICCs. In the urinary tract, including the renal pelvis, ureter, bladder, and urethra, KIT-positive ICCs, which are referred to as interstitial cells (IC), ICC-like cells, or myofibroblasts [11], have also been identified by their morphological characteristics [5, 12, 13], but show variability among tissues, which may account for individual characteristics of the organs [13]. 

Many groups have attempted to elucidate their physiological features in the bladder, but have shown that ICC in the bladder does not necessarily have the typical physiological function of ICC in the gastrointestinal tract [11]. Identifying the functions of ICCs may be a shortcut to clarify the pathophysiology of OAB and DO. Thus, in this review, we summarize the distribution and function of KIT-positive ICC in the bladder as well as the association between KIT-positive ICCs and OAB, and discuss the possible therapeutic target of KIT-positive ICC for OAB in the future.

## 2. Distribution and Morphology of KIT-Positive ICC 

ICC has immunoreactivity for vimentin, connexin-43, and cGMP [14–16]. In addition, vanilloid, purinergic, and muscarinic receptors were expressed on suburothelial ICCs [17–19]. On the other hand, KIT, which is expressed by ICC but not smooth muscle or fibroblasts [9, 10], is a well-established detection marker of ICC; however, definitive evidence remains lacking whether KIT was expressed in other structures in the bladder. Some researchers demonstrated that the presence of ICC in the urinary bladder has been demonstrated using antibodies to KIT (also known as c-kit) in rodents and humans. ICCs in the bladder are located throughout the bladder wall [20] and can be divided into at least two subpopulations by their morphology and orientation, that is, ICCs in detrusor smooth muscle layers (detrusor ICCs) and ICCs in the suburothelial layers (suburothelial ICCs) [15, 20, 21]. These ICCs are closely associated with detrusor smooth muscles and make structural interactions with cholinergic nerves in each region [15, 20–22].

In the detrusor smooth muscle layer, ICCs are preferentially located along the boundary of smooth muscle bundles and are also distributed between muscle bundles. They run in parallel with the smooth muscle bundles and are closely associated with intramural nerves [20]. These morphological findings suggest that ICCs in the bladder may act as pacemakers like those in the gastrointestinal tract as well as playing an important role in cell-to-cell communication to integrate signals in the bladder wall, although the hypothesis of the role of ICCs in the urinary bladder as pacemaker cells has remained controversial.

**Figure 1:**
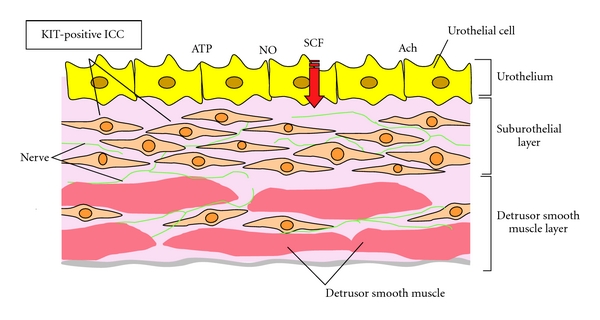
Distribution and morphology of Kit-positive suburothelial and detrusor ICCs and interaction with urothelium, nerves, and smooth muscle.

Suburothelial ICCs, which are also referred to as myofibroblasts, have a spindle- and stellate-shaped morphology with several branches emanating from a central soma [11, 20–23]. They are extensively linked by gap junctions to form a functional syncytium [15]. In addition, double-labeling with KIT and a nerve detection marker, PGP9.5, demonstrated that ICCs are in close apposition to one another and nerves, and form a interconnected cellular network [14], supposedly involved in signaling pathways of the bladder, and may play a role in moderating the sensory process, leading to the initiation of the micturition reflex [15] (Figure 1).

**Figure 2:**
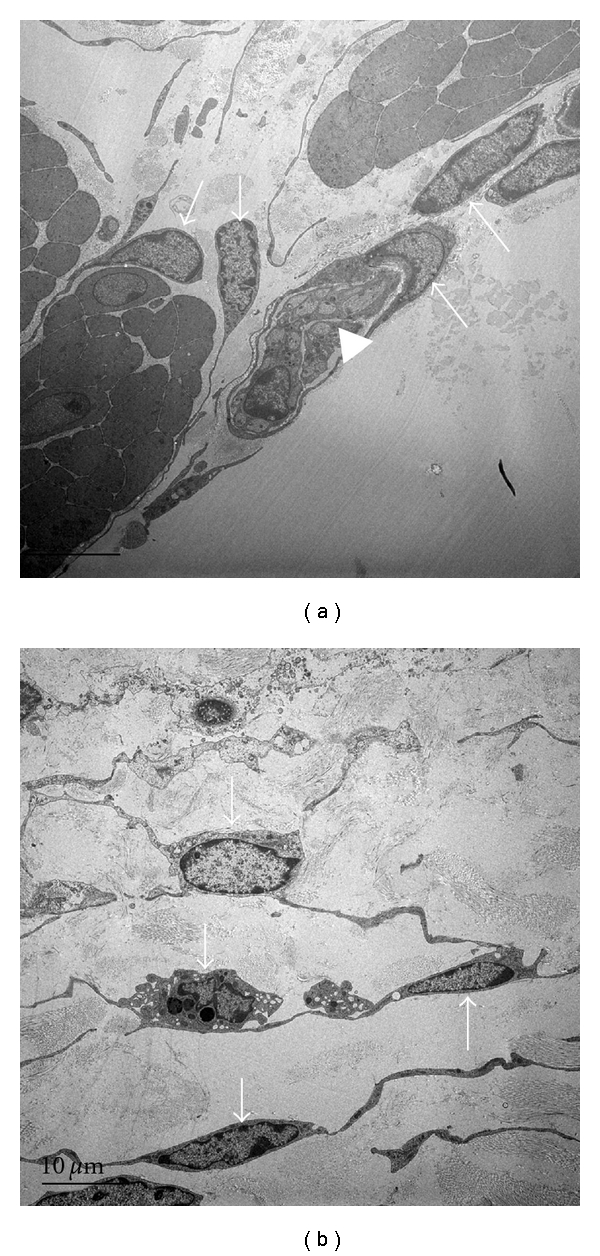
Electron micrographs of ICC-LC in the guinea-pig bladder. ICC-LCs made close contact with nerves and each other. Arrow: ICC, arrowhead: nerves, ×4,000.

ICCs in the bladder were also distinguishable from other cells by their unique ultrastructural features. There are several reports of ultrastructural characterization of ICC observed by transmission electron microscopy [6, 14, 23, 24]. A fundamental feature of ICCs is spindle- or stellate-shaped cells with pale eosinophilic cytoplasm and an elongated electron-dense nucleus (Figure 2). A critical element in the ultrastructure of these cells is the fibronexus, a cell-to-matrix junction, consisting of myofilament and fibronectin filament systems converging on a discrete cell-surface plaque [24]. Rasmussen et al. reported detailed information of the ultrastructure of detrusor ICCs, and revealed two different types of ICC in the human detrusor smooth muscle layer: a CD34-positive, CD-117-negative cell with a slender cytoplasmic process and myoid features, and a fibroblast-like cell. They concluded that detrusor ICCs may be analogous to ICC in the gastrointestinal tract. Wiseman et al. reported the characteristics of suburothelial ICCs [16], showing a layer of cells with the cytological characteristics of both fibroblasts and smooth muscle cells, and that ICCs included bundles of fine cytoplasmic filaments, dense bodies, linear arrays of subsurface vacuoles, and the presence of an interrupted basal lamina. These cells had close contact with unmyelinated axonal varicosities containing a mixture of clear and large dense-cored vesicles, or clear vesicles alone. Johnston et al. have also shown the ultrastructural profile of ICCs, including an absent thick filament, dense bodies or dense bands typical of smooth muscle cells, and mitochondria, ribosomes, vesicles, Golgi, and a well-developed nondilated rER [23]. These morphological studies provide a basis for future morphological and physiological investigations of ICCs under conditions of impaired bladder function.

## 3. Function of Kit-Positive ICC

ICCs in the gastrointestinal tract are known to be the origin of pacemaker signals that underlie their spontaneous activity, and they have a major role in the transmission of signals from enteric neurons to smooth muscle cells [25, 26]. Similarly, the bladder develops phasic or autonomous activity consisting of rhythmical transient contractions during the filling phase [27, 28]; however, single myocytes can generate action potentials, but not spontaneously [27]. Hashitani et al. reported that spontaneous action potentials and associated calcium waves occur almost simultaneously along the boundary of bladder smooth muscle bundles and then propagate to the other boundary, probably through gap junctions [29]. Recent evidence suggests that ICCs in the bladder may also play a role in determining the pattern of spontaneous activity, although their precise role is less well established in the urinary tract than in the gastrointestinal tract [30–32]. Therefore, one of the main focuses of some researchers is to identify the role of ICCs as either pacemaker cells to drive the smooth muscle wall or as intermediaries in neuromuscular transmission in the bladder.

Whether ICCs can act as pacemaker cells remains controversial. As described above, in the detrusor muscle layer, ICCs are found preferentially at the boundary of muscle bundles from where spontaneous Ca^2+^ transients originate, suggesting that they may be crucial in generating spontaneous excitation [21]. On the other hand, Hashitani et al. reported that spontaneous Ca^2+^ transients recorded from ICCs in fact occurred independently of those of smooth muscles even when synchronous Ca^2+^ waves swept across muscle bundles [33]. ICCs may be more important in mediating the propagation of action potentials along the bundles than in actually generating them [27, 29]. The finding in the human bladder that c-kit labeling showed significantly more ICCs in human OAB detrusor than in normal specimens [34] may support this suggestion, since in these tissues the contractions appear better coordinated across the strips [20]; therefore, they may not be electrical pacemaker cells. 

Recently, it has been suggested that ICCs may function as sensing network receiving/sending signals from/to the urothelium, modulating afferent bladder innervations, and/or activating a spinal or intramural reflex arc [35]. Sui et al. demonstrated that suburothelial ICCs respond to exogenous agents implicated in modulating bladder sensory responses; responses augmented by physical intercellular contact [36]. Not only detrusor ICCs but also suburothelial ICCs show spontaneous electrical and Ca^2+^ signaling [22, 23, 33, 37, 38]. They also respond to exogenous application of neurotransmitters such as adenosine triphosphate (ATP) and acetylcholine (Ach), and express purinergic (P2Y_6_), cholinergic (M_3_) receptors, and prostaglandin receptor types 1 and 2 (EP1 and EP2) [18, 19, 23, 39]. These findings suggest that ICCs act in the sensory processes of the bladder by responding to ATP, Ach, and prostaglandin, and might play an important functional role in the control of bladder function. In addition, other transmitters, such as connexin-43 (gap junctions, e.g., intercellular communication) and cGMP (responds to nitric oxide; NO) expressed by ICCs, are probably very important for normal and pathologic (OAB) physiology [15, 29].

Although KIT is used as an identification marker of ICCs, recent reports have suggested that KIT is not only a detection marker of these cells, but also may play a crucial role in the control of bladder function [34, 40]. Several reports have evaluated the role of KIT using KIT mutant mice and rats [41–43]. McCloskey et al. have recently reported the physiological function of ICC-like cells in the bladder using heterozygous KIT mutant mice (W/Wv), which have a point mutation at amino acid 660 in *c-kit* that causes a reduction but not abolition of tyrosine kinase activity. These mice had KIT- and vimentin-immunopositive ICC, and there are similarities in the electrical and contractile properties of W/Wv and wildtype detrusors [44]. On the other hand, homozygous KIT mutant WsRC Ws/Ws rats, which have a 12-base deletion in the tyrosine kinase domain of c-kit cDNA rats [41], have impaired pacemaker activity in the ileum and colon, which induced movement disturbance [42, 43]. We investigated morphological and physiological findings in the bladder of KIT mutant rats in order to clarify whether disturbance of the KIT pathways affects bladder activity [45]. Each parameter of cystometry in KIT mutant rats was similar to that of wildtype rats under normal conditions. Interestingly, however, the reduction in intercontraction intervals in KIT mutant rats with chemical cystitis was smaller than in wildtype rats, suggesting reduced noxious bladder sensations in KIT mutant rats. These results indicate that KIT plays an important role in bladder function, especially under pathological conditions, and certain voiding disturbances may be associated with impaired KIT signaling in ICCs.

Stem cell factor (SCF), a natural ligand for KIT, is associated with various biologic phases, such as hematopoiesis, reproduction, regeneration, and cell proliferation [46]; however, the distribution and role of SCF in the urinary bladder remains unknown, although the role of c-kit in the urinary bladder has been gradually clarified. Our preliminary data suggest that SCF produced in the urothelium of the urinary bladder may act as a possible mediator by binding to c-kit [47]. The SCF/c-kit pathway leads to the activation of multiple pathways, including phosphatidyl-inositol-3 kinase, phospholipase C-gamma, Src kinase, Janus kinase/signal transducers, and activators of transcription and mitogen-activated protein kinase pathways [48]. Recognition of the biological properties and elucidation of the mechanism of the SCF/c-kit pathway in the bladder may provide more insight into the physiology of the bladder.

## 4. KIT-Positive ICC and Pathophysiology of OAB and DO

Human gastrointestinal motility disorders, such as gastroparesis, chronic idiopathic intestinal pseudoobstruction, achalasia, and chronic constipation, have been associated with loss of ICC in dysfunctional regions of the gastrointestinal tract [49]. Although little is known about the role of ICCs in the bladder, the present knowledge suggests that the functions of ICCs may be region-specific, particularly under pathological conditions [50]. There have been reports of the correlation between ICCs in the bladder and OAB or DO. Biers et al. demonstrated that c-kit-positive ICCs are more numerous in human OAB detrusor than normal detrusor [34], suggesting that detrusor ICC is associated with the pathophysiology of OAB. Under OAB conditions, increased electrical coupling between smooth muscle cells may account for enhanced excitability of detrusor smooth muscles [51]. Thus, spontaneous excitation resulting from spontaneous action potentials [52] may spread for a longer distance and cause synchronous contractions of multiple muscle bundles to elevate intravesical pressure [13]. Indeed, micromotions of the bladder wall, which may be attributed to spontaneous contractions of a unit of muscle bundles, have been reported to be enhanced in a rat model of bladder overactivity [53]. 

Although the urinary bladder urothelium has classically been thought of as a passive barrier, recent studies have demonstrated that the urothelium is involved in sensory mechanisms and releases several bioactive mediators, such as ATP, nitric oxide, and acetylcholine. Although a neurogenic basis has been considered for the changes in both efferent and afferent autonomic nerves, the role of increased signal transmission from the urothelium to afferent nerves via suburothelial ICCs during the micturition reflex has attracted particular considerable attention [54]. Therefore, several researchers have focused on the correlation between suburothelial ICCs and the pathophysiology of OAB and DO, because they may play an important role in signal transmission and be responsible for bladder control. We investigated the distribution of ICCs in guinea-pigs with partial bladder outlet obstruction (PBOO), which showed bladder overactivity in cystometry [55]. The population of KIT or vimentin immunoreactive ICCs was increased in subserosal layers and their distribution was altered in the suburothelial layer in PBOO bladders, suggesting that the altered distribution of ICCs may contribute to the pathophysiology of bladder overactivity. Therefore, not only the disturbance of spontaneous contractility caused by altered ICC signal transduction between nerves and smooth muscle cells in the detrusor smooth muscle layer but also the disturbance of signal transduction between urothelial cells and sensory nerves via suburothelial ICC may induce OAB and DO. In addition, ultrastructural features of ICC changed in the PBOO model. This may produce abnormal signal transduction between ICC and the nerves or smooth muscle cells [55], suggesting that quantitative or qualitative changes in ICCs may account for the pathologically increased signal transmission between either homogenous or heterogeneous populations of cells in the bladder wall; however, since the pathophysiology of human OAB is not necessarily consistent with pBOO-induced detrusor overactivity, further study using human OAB specimens will be needed.

## 5. KIT-Positive ICC as a Therapeutic Target for OAB in the Future

Normal physiological contraction of the urinary bladder is predominantly mediated by muscarinic receptors, primarily the M_3_ subtype, with the M_2_ subtype playing a secondary backup role. On the other hand, bladder relaxation seems to be mediated by *β*-adrenoceptors, in most species involving a strong *β*_3_ component; therefore, interference with the signal transduction of these receptors may be a viable approach to develop drugs for the treatment of OAB [56]. It is well established that antimuscarinic drugs are effective in reducing symptoms and improving the quality of life of patients with OAB. Currently, antimuscarinic drugs are the first choice in the pharmacological treatment of OAB. Besides their status as the current standard of care, compliance and persistence are often affected by adverse effects. Although selective *β*_3_-adrenoceptor agonists are potentially useful agents for treating OAB, other options of medical treatment for OAB will be needed. 

Imatinib mesylate (Glivec) is a selective inhibitor of c-kit receptor tyrosine kinase and the oncogene Bcr-Abl, and has Food and Drug Administration approval for the treatment of chronic myeloid leukemia and gastrointestinal stromal tumor. Several researchers have demonstrated that inhibition of c-kit reduced bladder activity via c-kit receptor on bladder ICCs [15, 34, 35, 40, 57]. We examined the effects of imatinib mesylate on spontaneous excitation and ion channel activity in detrusor smooth muscles of the guinea-pig bladder using intracellular microelectrodes, isometric muscle tension recordings and patch clamp techniques [57]. Imatinib mesylate (10 microM) converted action potential bursts into continuous firing without affecting their shape but at 50 microM abolished spontaneous action potentials in single smooth muscle cells. It had little effect on inward and outward currents at <10 *μ*M, but inhibited them at >50 *μ*M. We also investigated the effects of imatinib mesylate on intravesical pressure of isolated guinea-pig bladders using whole organ bath techniques, and demonstrated that imatinib mesylate reduced the amplitude of spontaneous pressure rises in the whole bladder in a dose-dependent manner [40]. The results suggest that ICC-like cells may be responsible for generating bursts of action potentials and contractions in detrusor smooth muscle. Biers et al. demonstrated that imatinib mesylate inhibited evoked smooth muscle contraction and spontaneous activity in human OAB detrusor, with less effect on normal human tissue [34]. They also demonstrated that imatinib mesylate improved bladder capacity, compliance, voided volume, urinary frequency and reduced contraction thresholds and spontaneous activity during guinea pig cystometry [34]. Vahabi et al. have recently reported that imatinib mesylate decreased the amplitude and frequency of carbacol-induced phasic contractions in both normal and diabetic tissues in a dose-dependent manner [35]. These reports showed that imatinib mesylate inhibited spontaneous contraction and, as a result, probably reduced OAB symptoms. On the other hand, as described above, bladder suburothelial ICCs may modulate both sensory responses from the bladder wall and spontaneous activity. Sui et al. showed that several responses that influenced bladder activity either directly or through activation of the sensory mechanism were significantly augmented by physical connections between adjacent cells, and such augmentation was abolished by imatinib mesylate [36]. They also found that imatinib mesylate reduced spontaneous contractile activity in the isolated bladder. Although more data about the potential of ICC as target cells will be needed before the clinical implications of these findings are elucidated, KIT-positive ICCs may be one of the therapeutic target cells to reduce bladder overactivity and blocking c-kit receptor may offer a new therapeutic strategy for OAB treatment in the future.

## 6. Conclusions

The current limitations of improving OAB therapies arise from our lack of knowledge regarding the primary pathophysiology of this disease. Clarifying the role of ICC function in the bladder may lead to greater understanding of the mechanisms of OAB, and provide a novel therapeutic target. KIT-positive ICCs may be involved in signal transmission, between smooth muscle bundles, from efferent nerves to smooth muscles and from urothelium to afferent nerves, and thus could be a crucial target for the pharmacological treatment of OAB and DO in the future; however, since the role of KIT in the urinary bladder is still not fully clarified, further investigation and more evidence will be needed.

## Abbreviations

ICC: Interstitial cells of Cajal
OAB: Overactive bladder
DO: Detrusor overactivity
ATP: Adenosine triphosphate
SCF: Stem cell factor.
